# Do ferrous iron-oxidizing acidophiles (*Leptospirillum* spp.) disturb aerobic bioleaching of laterite ores by sulfur-oxidizing acidophiles (*Acidithiobacillus* spp.)?

**DOI:** 10.3389/fmicb.2024.1359019

**Published:** 2024-04-09

**Authors:** Stefanie A. Hetz, Axel Schippers

**Affiliations:** Federal Institute for Geosciences and Natural Resources (BGR), Hannover, Germany

**Keywords:** bioleaching, reductive dissolution, laterite, *Acidithiobacillus*, *Leptospirillum*, nickel, cobalt

## Abstract

The extraction of nickel, cobalt, and other metals from laterite ores via bioleaching with sulfur-oxidizing and ferric iron-reducing, autotrophic, acidophilic bacteria (e.g. *Acidithiobacillus* species) has been demonstrated under anaerobic as well as aerobic conditions in experiments in different laboratories. This study demonstrated the bioleaching of laterites from Brazil with the addition of elemental sulfur in 2-L stirred-tank bioreactors with pure and mixed cultures of *Acidithiobacillus* and *Sulfobacillus* species under aerobic conditions. In particular, a potential disturbance of mineral dissolution under aerobic conditions by ferrous iron-oxidizing acidophiles likely introduced as contaminants in an applied bioleaching process was investigated with *Leptospirillum ferrooxidans* at 30°C and *Leptospirillum ferriphilum* at 40°C, at maintained pH 1.5 or without maintained pH leading to an increase in acidity (with pH values <1.0) due to the biological production of sulfuric acid. Despite the proportion of ferrous iron to the total amount of extracted iron in the solution being drastically reduced in the presence of *Leptospirillum* species, there was a negligible effect on the extraction efficiency of nickel and cobalt, which is positive news for laterite bioleaching under aerobic conditions.

## 1 Introduction

The increasing demand for nickel and cobalt for battery production and other applications has led to increased mining of laterite ores. Pyrometallurgy is applied for saprolitic laterites but does not apply to limonitic laterites; here, hydrometallurgical processing options such as high-pressure acid leaching (HPAL) are chosen, which means high-energy consumption and processing costs (Stanković et al., 2020). Biohydrometallurgical processing of laterite ores promises lower energy consumption, lower acid consumption, and less expensive equipment due to relatively moderate acidic conditions over established laterite ore processing technologies.

Processing of limonitic laterites via bioleaching has been studied for about a decade (Roberto and Schippers, 2022; Santos and Schippers, 2023). Most studies focused on acidophiles that couple the oxidation of added elemental sulfur to sulfuric acid with the reduction of ferric iron in laterite mineral phases leading to a dissolution of particular mineral phases and solubilization of nickel and cobalt. The biogenic ferrous iron acts as a chemically reducing agent here (Stanković et al., 2022). First reports described a Ferredoxin process concept in which *Acidithiobacillus (At.) ferrooxidans* reduces ferric iron under anaerobic conditions from goethite as a main mineral phase, and the term “reductive bioleaching” was introduced (du Plessis et al., 2011; Hallberg et al., 2011; Johnson and du Plessis, 2015). A later study showed that other Ni- and Co-bearing mineral phases in laterites such as Mn-rich mineral phases and magnesium silicates are dissolved, rather than goethite (Stanković et al., 2022). This and other previous studies (Marrero et al., 2015, 2017) showed that Ni and Co recovery from limonitic laterites via bioleaching under aerobic conditions with the aerobic sulfur-oxidizers *At. thiooxidans* and *At. caldus* was at least as efficient as bioleaching under anaerobic conditions with *At. ferrooxidans*, with the advantage of lower acid consumption and no need for a costly gassing with dinitrogen to maintain anaerobic conditions. Aerobic bioleaching of ferric iron-rich laterites was efficient at low pH < 1. Overall, the reaction mechanism for this aerobic process is still unknown. However, it has been hypothesized that ferric iron is reduced only chemically, by reduced sulfur compounds released into solution by the sulfur-oxidizing *Acidithiobacillus* (Marrero et al., 2020; Johnson et al., 2021). Supporting this hypothesis, hydrogen sulfide and thiosulfate were recently detected as such sulfur intermediates in ferric iron reduction experiments with acidophiles (Breuker and Schippers, 2024).

A potential problem with aerobic bioleaching of limonitic laterites is that acidophilic ferrous iron-oxidizers such as *Leptospirillum (L.) ferrooxidans* would grow as contaminants in industrial bioleaching operations, which cannot run under sterile conditions (Smith and Johnson, 2018). In this case, ferrous iron would be oxidized to ferric iron and the ferrous iron concentration in solution might be too low for an efficient reductive dissolution of laterite mineral phases with the reductant ferrous iron (Moro et al., 2023). To address this concern, this study presents a couple of aerobic, stirred-tank bioleaching experiments in which iron-oxidizing acidophiles (*Leptospirillum*) were added to sulfur-oxidizing acidophiles (*Acidithiobacillus*) in co-culture, and the bioleaching efficiency was evaluated.

## 2 Materials and methods

### 2.1 Laterite sample

A laterite ore sample was obtained from a stockpile of the Anglo-American-owned Barro Alto mine in the state of Goiás, Brazil. The physical properties, geochemistry, and mineralogy of the sample BaSt are comprehensively described elsewhere (Stanković et al., 2022). Briefly, the sample was ocher-colored, fine-grained clayey-silty material, completely decomposed and disaggregated. It could be categorized as a mixed limonitic-saprolitic laterite with different nickel- and cobalt-bearing mineral phases. The chemical composition included approximately 42% Fe_2_O_3_, 26% SiO_2_, 9.4% MgO, 4.8% Al_2_O_3_, 0.8% MnO, 15.8 g/kg Cr, 13.6 g/kg Ni, and 1.3 g/kg Co.

### 2.2 Laterite bioleaching experiments

Bioleaching was carried out on a laboratory scale in 2-L stirred-tank bioreactors, containing 1.5 L of a basalt salts medium with trace elements (Ňancucheo et al., 2016) with an initial pH of 1.5, 1% (w/v) elemental sulfur, and 36 mM ferrous iron. Bioreactors were constantly stirred and supplied with compressed air to ensure oxygen and CO_2_ supply. Pre-grown type strain pure cultures of sulfur-oxidizing *At. thiooxidans* DSM 14887^T^ and *At. caldus* DSM 8584^T^ were used to evaluate the bioleaching efficiency in the absence of iron-oxidizers. To evaluate bioleaching efficiency in the presence of iron-oxidizers, type strains of *L. ferrooxidans* DSM 2705^T^ and *L. ferriphilum* DSM 14647^T^ were mixed with either *At. thiooxidans* or *At. caldus*, respectively (1:1, mixed cultures). Temperature was kept constant at 30°C for *At. thiooxidans* ± *L. ferrooxidans* and at 40°C for *At. caldus* ± *L. ferriphilum*. In addition, bioleaching efficiency was tested with artificial consortia consisting of different sulfur- and iron-oxidizers at 30°C and 40°C (Table 1) to get closer to non-sterile industrial conditions, mimicking possible contamination with multiple microorganisms. All bioreactors were inoculated with 10% (v/v) pre-grown pure or mixed cultures and run for 4 days for further growth. Afterward, 10% (w/v) laterite was added to start bioleaching. During 15 days of bioleaching, the pH was adjusted with 1 M H_2_SO_4_ or 1 M NaOH to be maintained at 1.5, or there was no pH maintenance. Samples for chemical and microbiological analyses were regularly taken. All experiments were performed in two replicate bioreactors if not stated otherwise.

**Table 1:** Mesophilic and moderately thermophilic, acidophilic bacteria used for bioleaching experiments in the two consortia at 30°C and 40°C, respectively.

Bacterium, DSM number	Consortium*	Fe(II)-oxidation^1,2^	S^0^-oxidation^1,2^	Growth^1,2,3^	Fe(III)-reduction^1,2,3^
*Acidithiobacillus caldus* 8584^T^	T	−	+	Aerobic	+
*Acidithiobacillus ferridurans* 29468^T^	M	+	+	Facultative anaerobe	+
*Acidithiobacillus ferriphilus* 100412^T^	M, T	+	+	Facultative anaerobe	+
*Acidithiobacillus ferrooxidans* 14882^T^	M	+	+	Facultative anaerobe	+
*Acidithiobacillus thiooxidans* 14887^T^	M	−	+	Aerobic	+
*Acidithiobacillus thiooxidans* 9463	M	−	+	Aerobic	+
*Leptospirillum ferriphilum* 14647^T^	T	+	−	Aerobic	−
*Leptospirillum ferrooxidans* 2705^T^	M	+	−	Aerobic	−
*Sulfobacillus acidophilus* 10332^T^	T	+	+	Facultative anaerobe	+
*Sulfobacillus benefaciens* 19468^T^	M, T	+	+	Facultative anaerobe	+
*Sulfobacillus harzensis* 109850^T^	M, T	+	+	Facultative anaerobe	+
*Sulfobacillus sibiricus* 17363^T^	M, T	+	+	Facultative anaerobe	+
*Sulfobacillus thermosulfidooxidans* 9293^T^	T	+	+	Facultative anaerobe	+
*Sulfobacillus thermotolerans* 17362^T^	M, T	+	+	Facultative anaerobe	+

*Indicates which consortium the microorganism was part of in the bioreactor runs; M = mesophilic (30°C); T = moderately thermophilic (40°C); for more information on iron and sulfur oxidation, growth, metabolism, and at what conditions ferric iron reduction occurs see: ^1^Malik and Hedrich (2022); ^2^Schippers (2007); ^3^Sarkodie et al. (2022).

### 2.3 Analytical methods and statistics

Liquid samples were analyzed for dissolved metals via inductively coupled plasma optical emission spectroscopy (ICP-OES). Ferrous iron and total iron concentrations were measured by colorimetric assays (Stookey, 1970). The pH and redox potentials (platinum-silver/silver chloride electrodes with results converted corresponding to the standard hydrogen electrode) were measured with electrodes (Blue Line, Xylem Analytics Germany Sales GmbH & Co. KG, Achalaich, Germany) and the Calimatic 766 Laboratory Meter (Knick Elektronische Messgeräte GmbH & Co. KG, Berlin, Germany). SYBR Green staining was used to determine cell numbers in liquid samples via fluorescence microscopy (Hedrich et al., 2016). Statistical analyses were performed in SigmaPlot version 12.3 (Systat Software, Inc., San Jose, CA, USA). Prior to statistical tests, basic data analyses were performed, including visual inspection of all measured variables coupled with the Shapiro–Wilk normality test. Analysis of variance (ANOVA) was used to test for the treatment effect, that is, differences between temperature and pH.

## 3 Results

### 3.1 Physiological data of laterite bioleaching experiments (pH, redox potential, and iron concentration)

#### 3.1.1 Laterite bioleaching at 30°C with *Acidithiobacillus thiooxidans* in the presence or absence of *Leptospirillum ferrooxidans*

**Figure 1:**
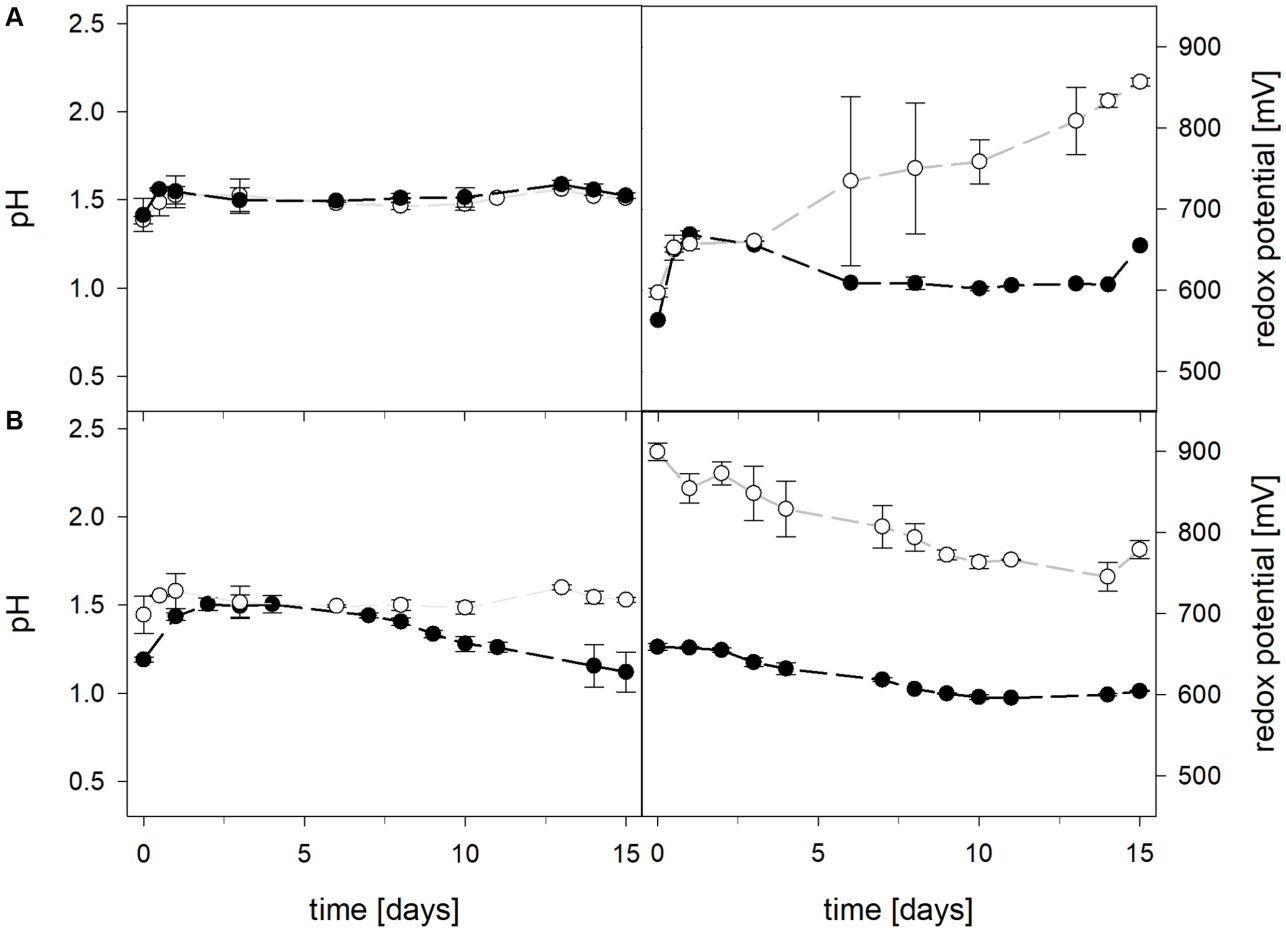
pH (left) and redox potential (right) during laterite bioleaching with a pure culture of *At. thiooxidans* (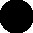) and a mixed culture of *At. thiooxidans* and *L. ferrooxidans* (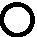) over 15 days under oxic conditions in 2-L stirred-tank reactors at maintained pH 1.5 **(A)** or not maintained pH **(B)**. Error bars show standard deviations from the mean values for two parallel bioreactor runs, except for *At. thiooxidans* and *L. ferrooxidans* at maintained pH 1.5 (*n* = 3).

**Figure 2:**
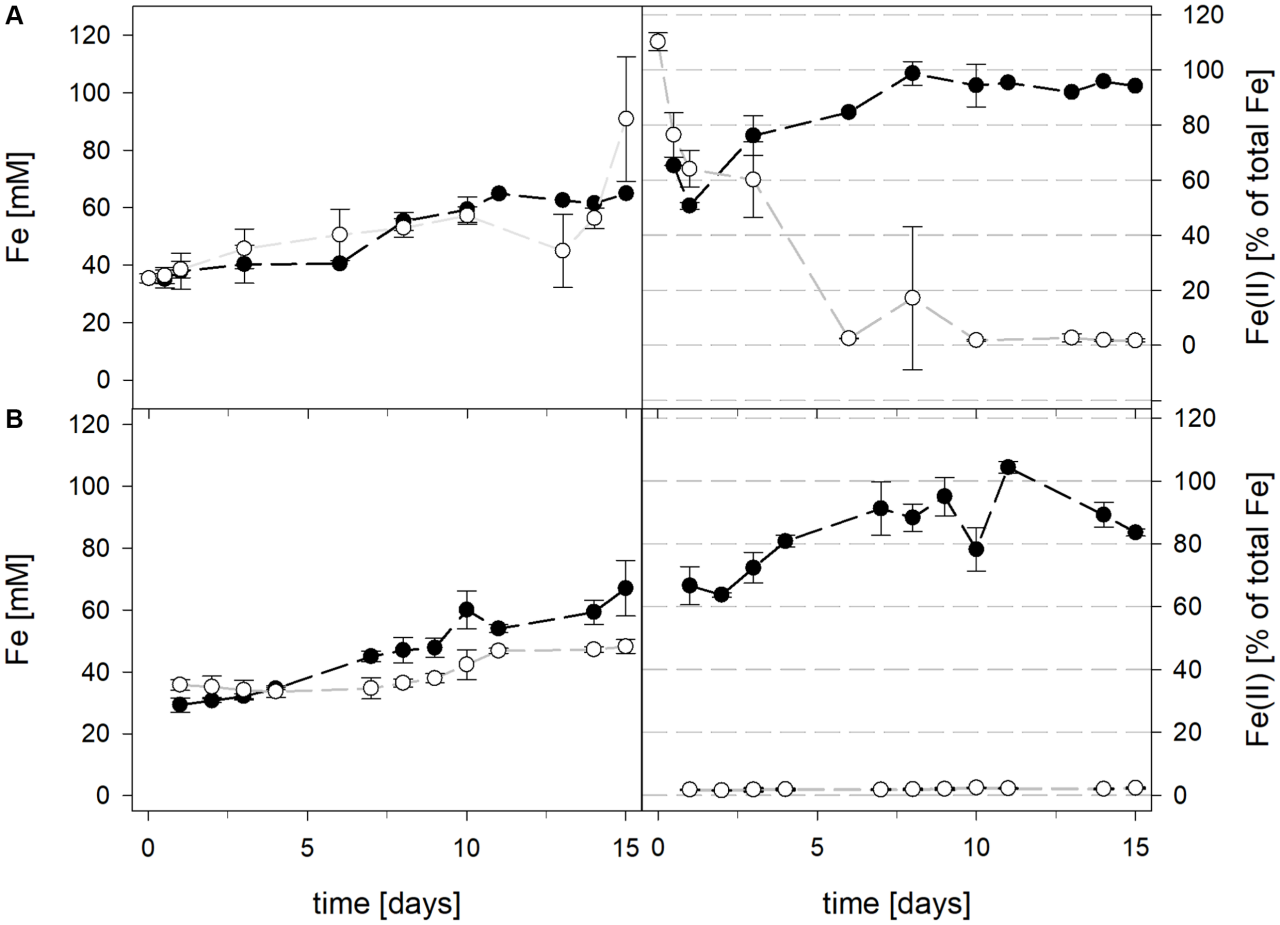
Total iron content (left) and ferrous iron as a percentage of total iron (right) during laterite bioleaching with a pure culture of *At. thiooxidans* (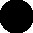) and a mixed culture of *At. thiooxidans* and *L. ferrooxidans* (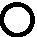) over 15 days under oxic conditions in 2-L stirred-tank reactors at maintained pH 1.5 **(A)** or not maintained pH **(B)**. Error bars show standard deviations from the mean values for two parallel bioreactor runs, except for *At. thiooxidans* and *L. ferrooxidans* at maintained pH 1.5 (*n* = 3).

Mean solution pH values during the pH-maintained bioleaching experiments with the *At. thiooxidans* pure culture and the *At. thiooxidans*–*L. ferrooxidans* mixed cultures were 1.52 ± 0.1 and 1.49 ± 0.1, respectively (Figure 1). In bioreactors containing the *At. thiooxidans*–*L. ferrooxidans* mixed culture without maintained pH, the pH was similar, with a mean pH of 1.52 ± 0.1. For the pure culture of *At. thiooxidans,* the pH dropped during incubation and reached 1.12 ± 0.1 after 15 days and was significantly different from the mixed culture experiments (*p* = 0.001). The redox potential in pure culture bioreactors with and without maintained pH behaved similarly (621 ± 32 mV and 622 ± 25 mV, respectively), whereas the redox potential of mixed cultures was considerably higher, reaching 857 ± 5 mV at adjusted maintained pH and 779 ± 11 mV when pH was not maintained (Figure 1). Disregarding whether the pH in the bioreactors was maintained or not, the differences in redox potential between pure and mixed cultures were significant with *p* = 0.005 and *p* < 0.001, respectively. Total iron concentration in the solution reached 29.8 ± 2.2 mM and 54.5 ± 13.4 mM in pure and mixed cultures, respectively, when pH was maintained (Figure 2). When pH was not maintained total iron in solution reached 37.8 ± 4.7 mM and 12.4 ± 0.4 mM in pure and mixed cultures, respectively. Ferrous iron as a percentage of total iron in solution behaved very differently in pure and mixed cultures, independent of pH. In all *At. thiooxidans* cultures, most of the total iron was present as ferrous iron, 98.7 ± 4.3% and 100 ± 1.8% when pH was maintained or not, respectively (Figure 2). In mixed cultures of *At. thiooxidans* and *L. ferrooxidans,* the ferrous iron proportion dropped quickly and at the end of experiments only represented 1.8 ± 0.6% and 2.3 ± 0.2% of total iron when pH was maintained or not, respectively. Differences in the amount of ferrous iron as a percentage of total iron were significantly different for pH 1.5 (*p* < 0.001) and not maintained pH (*p* < 0.001) between pure and mixed cultures.

#### 3.1.2 Laterite bioleaching at 40°C with *Acidithiobacillus caldus* in the presence or absence of *Leptospirillum ferriphilum*

**Figure 3:**
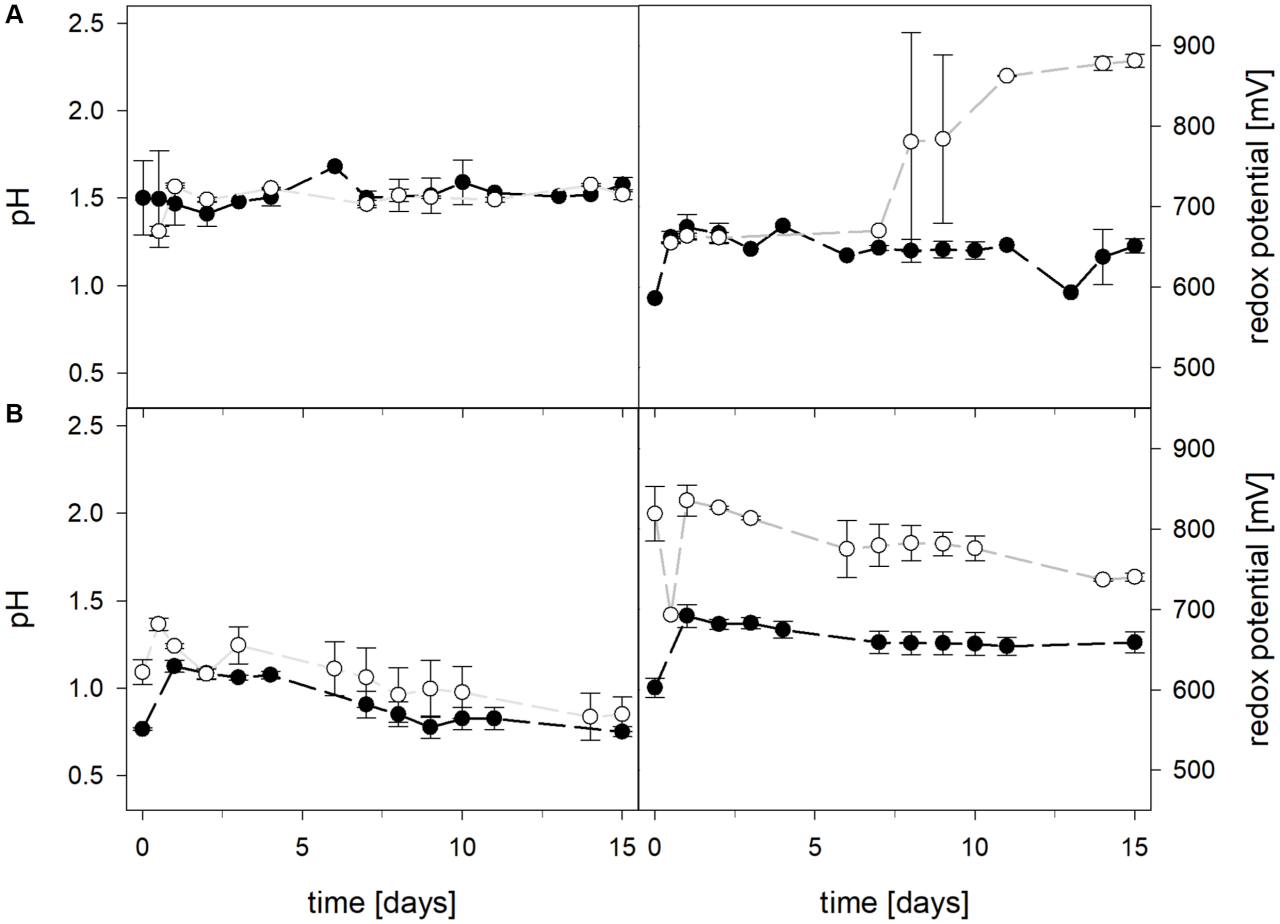
pH (left) and redox potential (right) during laterite bioleaching with a pure culture of *At. caldus* (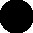) and a mixed culture of *At. caldus* and *L. ferriphilum* (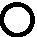) over 15 days under oxic conditions in 2-L stirred-tank reactors at maintained pH 1.5 **(A)** or not maintained pH **(B)**. Error bars show standard deviations from the mean values for two parallel bioreactor runs.

**Figure 4:**
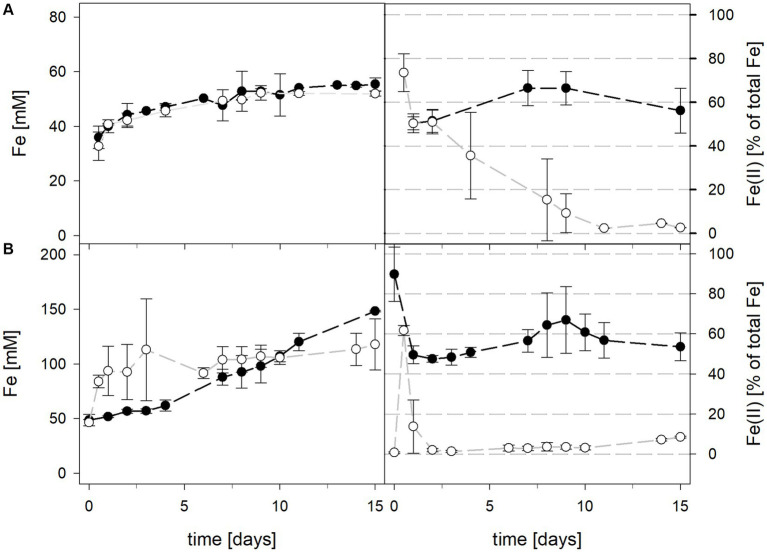
Total iron content (left) and ferrous iron as a percentage of total iron (right) during laterite bioleaching with a pure culture of *At. caldus* (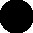) and a mixed culture of *At. caldus* and *L. ferriphilum* (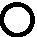) over 15 days under oxic conditions in 2-L stirred-tank reactors at maintained pH 1.5 **(A)** or not maintained pH **(B)**. Error bars show standard deviations from the mean values for two parallel bioreactor runs.

Mean solution pH values of *At. caldus* and *At. caldus*–*L. ferriphilum* during bioleaching at maintained pH were 1.52 ± 0.1 and 1.50 ± 0.1, respectively (Figure 3). When the pH was not maintained in the pure culture of *At. caldus* and mixed culture of *At. caldus* and *L. ferriphilum*, the pH behaved similarly and dropped below 1, reaching a minimum of 0.78 ± 0.0 in pure culture and 0.77 ± 0.1 in mixed culture, despite being significantly different throughout incubation (*p* = 0.012). The mean redox potential in pure culture at maintained pH was 649 ± 19 mV, and 668 ± 13 mV when the pH was not maintained (Figure 3). Again, the redox potential of the mixed culture was higher and climbed to 882 ± 8 mV at pH 1.5 and 773 ± 42 mV when pH was not maintained. For both maintained pH 1.5 and not maintained pH, the differences in redox potential between pure and mixed cultures were significant with *p* = 0.000 and *p* < 0.001, respectively. Total iron in solution was almost identical at maintained pH in pure and mixed cultures after 15 days reaching 19.5 ± 1.2 mM and 19.2 ± 3.1 mM, respectively. However, ferrous iron represented 94.1% of total iron in pure culture and only 1.8 ± 0.6% in mixed culture (Figure 4), and the difference between the two treatments was significant (*p* = 0.013). In bioreactors without maintained pH, total iron in solution reached 92.4 ± 5.0 mM in pure culture and 42.2 ± 9.7 mM in mixed culture, of which 59.1 ± 13.6% and 11.2 ± 2.0% were present as ferrous iron, respectively (Figure 4), presenting a statistically significant difference with *p* < 0.001.

#### 3.1.3 Laterite bioleaching with mesophilic or moderately thermophilic consortia of acidophiles in the presence or absence of *Leptospirillum* species

**Figure 5:**
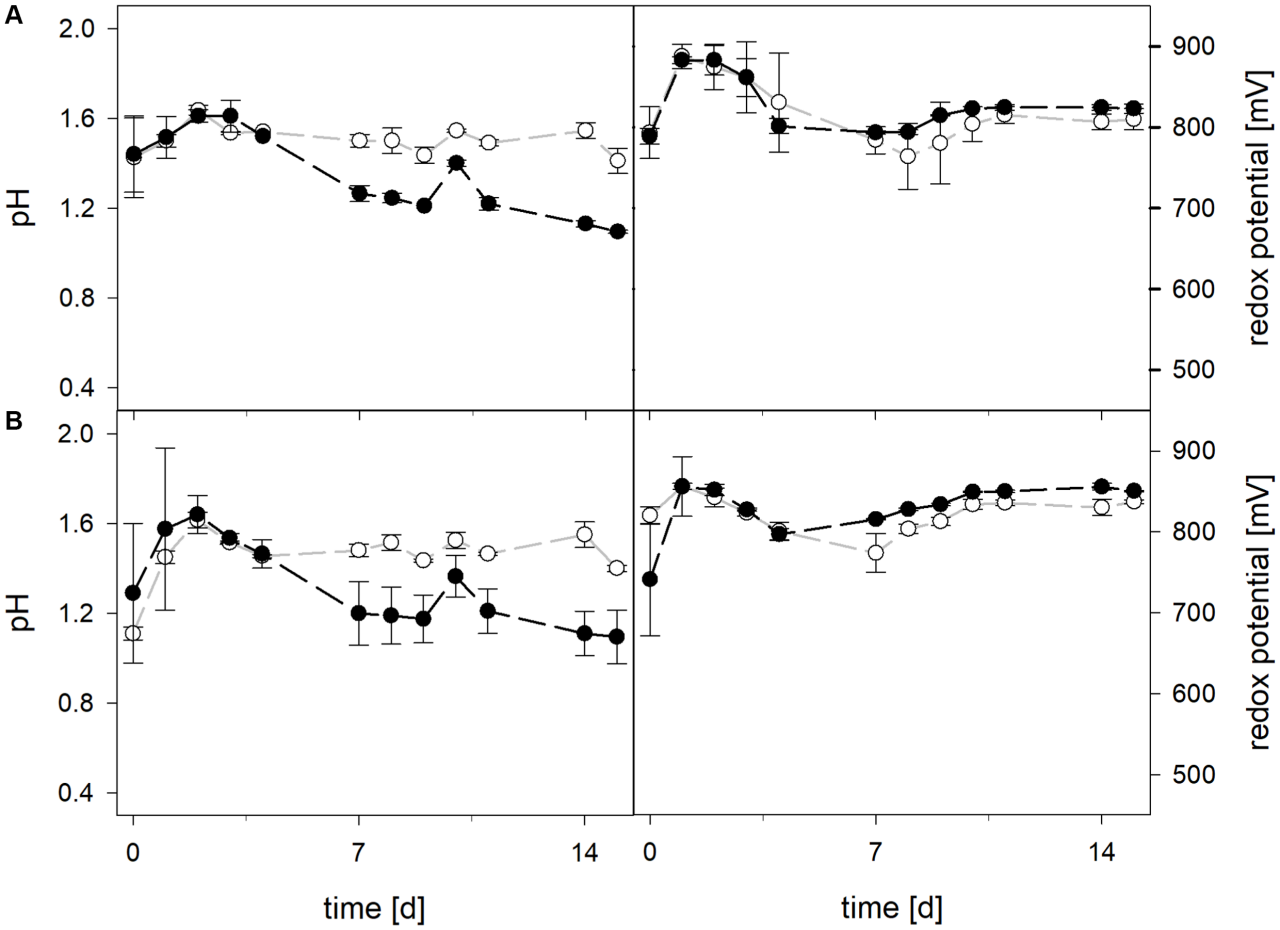
pH (left) and redox potential (right) during laterite bioleaching with consortia consisting of acidophilic sulfur- and iron-oxidizers at maintained pH 1.5 (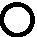) and not maintained pH (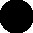) over 15 days under oxic conditions in 2-L stirred-tank reactors at 30°C **(A)** or 40°C **(B)**. Error bars show standard deviations from the mean values for three and two parallel bioreactor runs at maintained pH 1.5 and not maintained pH, respectively.

**Figure 6:**
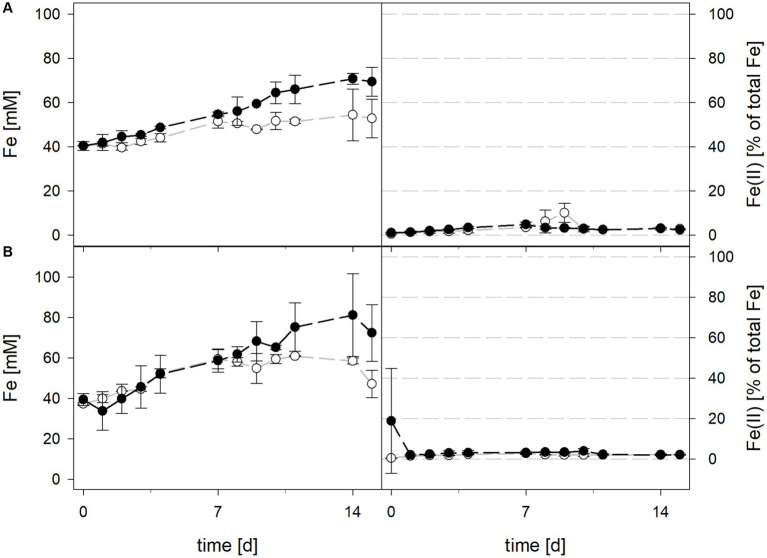
Total iron content (left) and ferrous iron as a percentage of total iron (right) during laterite bioleaching with consortia consisting of acidophilic sulfur- and iron-oxidizers at maintained pH 1.5 (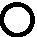) and not maintained pH (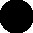) over 15 days under oxic conditions in 2-L stirred-tank reactors at 30°C **(A)** or 40°C **(B)**. Error bars show standard deviations from the mean values for three and two parallel bioreactor runs at maintained pH 1.5 and not maintained pH, respectively.

During bioleaching at maintained pH, the mean pH values of the 30°C and 40°C consortia were 1.50 ± 0.1 and 1.49 ± 0.1, respectively (Figure 5). When pH was not maintained in consortia bioreactors at 30°C and 40°C, the pH behaved similarly and dropped down during incubation, reaching 1.06 ± 0.2 at 30°C and 1.30 ± 0.1 at 40°C. Differences in pH between maintained and not maintained treatments with consortia were statistically significant at 40°C (*p* = 0.046), but not at 30°C. The mean redox potential at 30°C and maintained pH was 818 ± 39 mV, and 828 ± 31 mV when the pH was not maintained (Figure 5). The redox potential at 40°C was higher, though also in a similar range with mean values of 824 ± 23 mV at pH 1.5 and 838 ± 18 mV when pH was not maintained. Total iron concentration in solution was comparable at 30°C and 40°C after 15 days when pH was maintained (48.3 ± 6.3 mM and 51.6 ± 6.8 mM, respectively) and not maintained (57.7 ± 10.8 mM and 61.4 ± 15.8 mM, respectively) (Figure 6). The ferrous iron concentration was low during incubation and the mean values ranged between 2.4 and 3.3% of total iron in all reactors (Figure 6).

### 3.2 Cell numbers during laterite bioleaching

**Figure 7:**
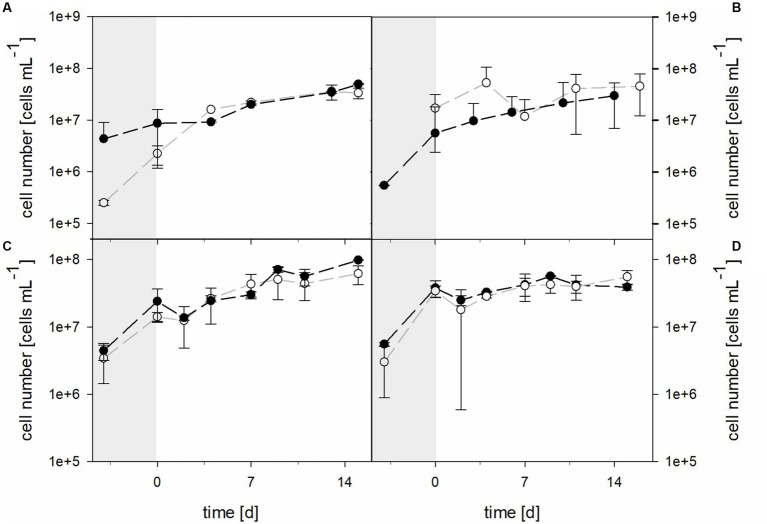
Cell numbers during pre-growth (shaded areas) and laterite bioleaching (days 0 to 14/15) with **(A)** *At. thiooxidans* (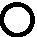) and *At. thiooxidans*–*L. ferrooxidans* (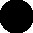) at not maintained pH and 30°C, **(B)** *At. caldus* (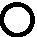) and *At. caldus*–*L. ferriphilum* (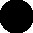) at not maintained pH and 40°C, and consortia consisting of acidophilic sulfur- and iron-oxidizers at maintained pH 1.5 (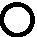) and not maintained pH (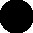) under oxic conditions in 2-L stirred-tank reactors at 30°C **(C)** or 40°C **(D)**. Error bars show standard deviations from the mean values for two parallel bioreactor runs.

Planktonic cell numbers were similar in 30°C bioreactors with the pure culture of *At. thiooxidans* and the mixed culture of *At. thiooxidans*–*L. ferrooxidans* when pH was not maintained (Figure 7). Bioreactors were inoculated with 2.51 × 10^5^ ± 2.96 × 10^4^ cells mL^−1^ of *At. thiooxidans* and 4.33 × 10^6^ ± 4.74 × 10^6^ cells mL^−1^ of the *At. thiooxidans*–*L. ferrooxidans* mixed culture. After 4 days of growth and before the addition of the laterite sample, bioreactors with pure culture contained 2.25 × 10^6^ ± 9.14 × 10^5^ cells mL^−1^, whereas bioreactors with mixed culture contained 8.64 × 10^6^ ± 7.47 × 10^6^ cells mL^−1^. Cell numbers increased in all bioreactors (with the initial difference in cell densities being reduced), reaching 3.35 × 10^7^ ± 7.68 × 10^6^ cells mL^−1^ in pure culture and 4.95 × 10^7^ ± 1.46 × 10^6^ cells mL^−1^ in mixed culture. Cell numbers for bioreactors with the mixed culture of *At. caldus* and *L. ferriphilum* started at 2.78 × 10^5^ ± 3.87 × 10^5^ cells mL^−1^ and reached 9.16 × 10^6^ ± 4.98 × 10^6^ cells mL^−1^ after 4 days, just before the addition of laterite (Figure 7). After 14 days of bioleaching by mixed cultures, the mean cell count reached 2.65 × 10^7^ ± 4.97 × 10^6^ cells mL^−1^. Unfortunately, cell numbers for *At. caldus* pure culture for inoculation of the bioreactors are not available due to technical reasons, but cell numbers had reached 1.95 × 10^7^ ± 1.71 × 10^6^ cells mL^−1^ before laterite was added (Figure 7). The cell counts showed some fluctuation during bioleaching and reached 3.97 × 10^7^ ± 8.65 × 10^6^ cells mL^−1^ after 5 days. The mean cell number of pure *At. caldus* culture and that of *At. caldus*–*L. ferriphilum* mixed culture was statistically significantly different (*p* = 0.024).

The cell numbers in consortia followed similar trends and showed no significant differences during laterite bioleaching at either 30°C or 40°C at maintained pH 1.5 or not maintained pH (Figure 7).

At 30°C the initial cell density was 3.42 × 10^6^ ± 1.99 × 10^6^ cells mL^−1^ in bioreactors with pH maintained at 1.5, while 4.44 × 10^6^ ± 1.28 × 10^6^ cells mL^−1^ were present in bioreactors without maintained pH. After 4 days of growth and before the addition of the laterite sample, bioreactors with pH 1.5 contained 1.41 × 10^7^ ± 2.15 × 10^6^ cells mL^−1^, whereas bioreactors without maintained pH contained 2.40 × 10^7^ ± 1.24 × 10^7^ cells mL^−1^. Cell numbers increased in all approaches, reaching 6.18 × 10^7^ ± 1.95 × 10^7^ cells mL^−1^ at pH 1.5 and 9.84 × 10^7^ ± 9.71 × 10^5^ cells mL^−1^ when pH was not maintained. Bioreactors running at 40°C with the moderately thermophilic consortium at maintained or not maintained pH had initial cell densities of 3.01 × 10^6^ ± 2.12 × 10^6^ and 5.56 × 10^6^ ± 3.34 × 10^5^ cells mL^−1^, respectively, and reached 3.42 × 10^7^ ± 6.64 × 10^6^ and 3.78 × 10^7^ ± 10.4 × 10^7^ cells mL^−1^ before the addition of the laterite sample (Figure 7). During bioleaching, the cell numbers did not change much and were at 5.55 × 10^7^ ± 1.30 × 10^7^ and 3.91 × 10^7^ ± 4.17 × 10^6^ cells mL^−1^ at the end of the runs at pH 1.5 and without maintained pH.

### 3.3 Extraction of nickel, cobalt, and iron in laterite bioleaching experiments

**Figure 8:**
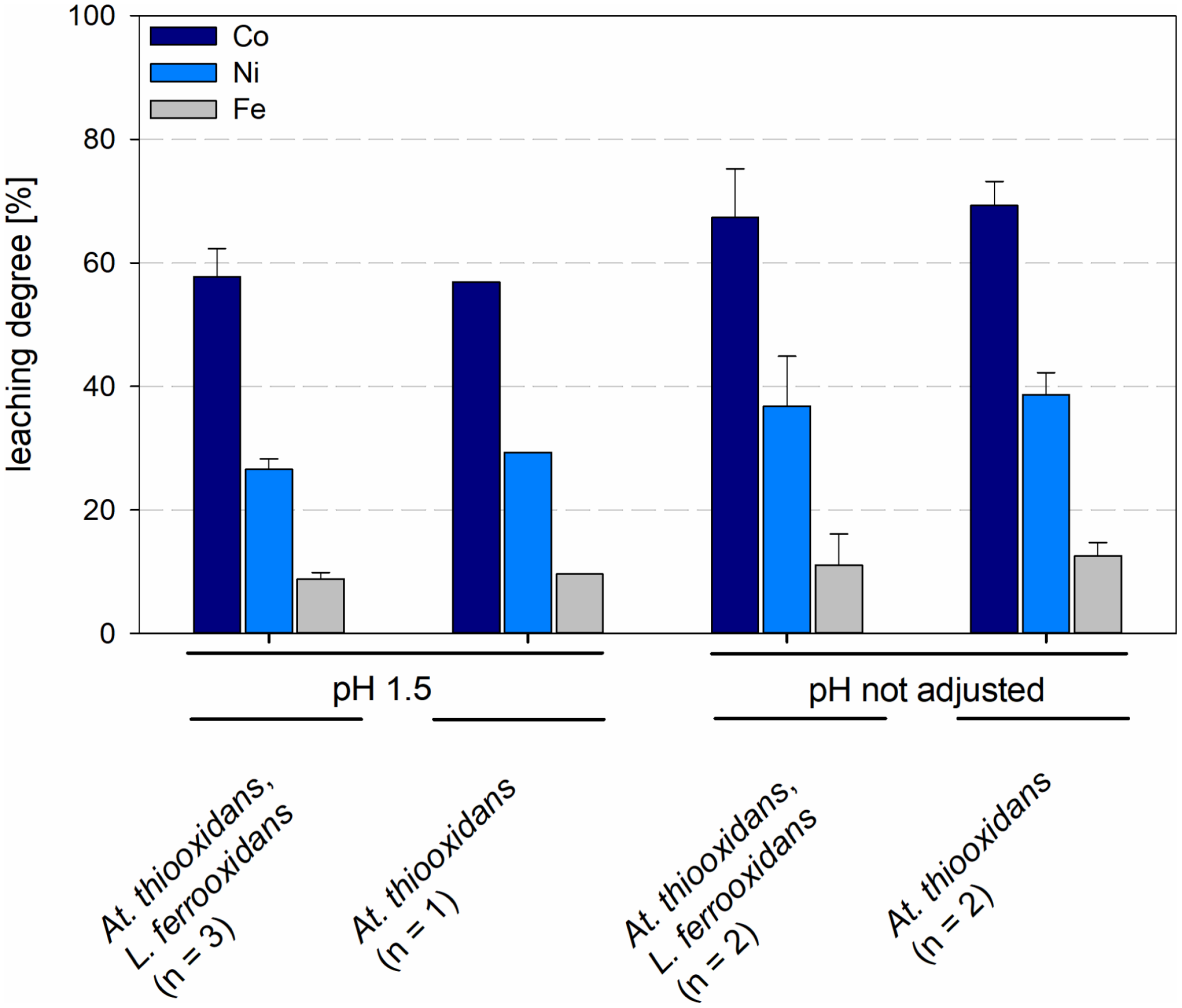
Leaching degree of Co, Ni, and Fe in laterite bioleaching experiments with a pure culture of *At. thiooxidans* and a mixed culture of *At. thiooxidans* and *L. ferrooxidans* after 15 days under oxic conditions in 2-L stirred-tank reactors at pH 1.5 or not maintained pH. Error bars show standard deviations from the mean values for n parallel bioreactor runs.

The leaching degree after 15 days with maintained pH for cobalt was 56.9% and for nickel 29.3% in *At. thiooxidans* pure culture and 57.8 ± 4.6% for cobalt and 26.5 ± 1.7% for nickel in mixed culture with *L. ferrooxidans*. When the pH was not maintained the leaching degrees for cobalt and nickel in pure culture were 69.3 ± 3.9% and 38.6 ± 3.6%, respectively, and 67.4 ± 7.8% and 36.7 ± 8.1% for cobalt and nickel, respectively, in mixed cultures (Figure 8). No statistically significant difference was detected between pure and mixed cultures or pH treatments.

**Figure 9:**
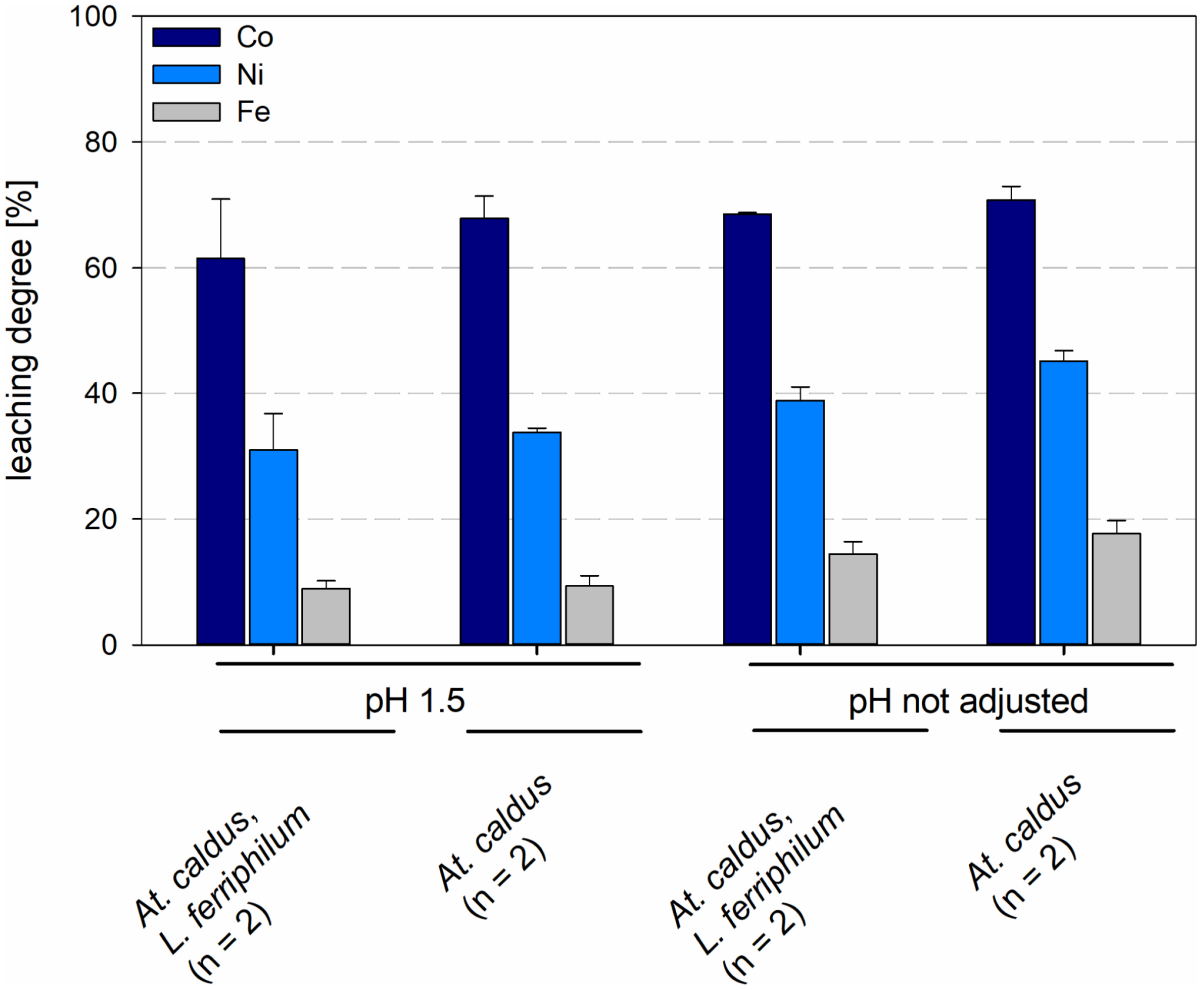
Leaching degree for Co, Ni, and Fe in laterite bioleaching experiments with a pure culture of *At. caldus* and a mixed culture of *At. caldus* and *L. ferriphilum* after 15 days under oxic conditions in 2-L stirred-tank reactors at pH 1.5 or not maintained pH. Error bars show standard deviations from the mean values for n parallel bioreactor runs.

Leaching degrees for cobalt and nickel were 67.8 ± 3.5% and 33.8 ± 0.7%, respectively, for *At. caldus* at pH 1.5 and 70.8 ± 2.2% and 45.2 ± 1.6%, respectively, for *At. caldus* at not maintained pH. Mixed cultures of *At. caldus* and *L. ferriphilum* at pH 1.5 showed leaching degrees of 61.5 ± 9.4% for cobalt and 31.0 ± 5.9% for nickel, when the pH was not maintained the leaching degrees were 68.5 ± 0.2% for cobalt and 38.8 ± 2.2% for nickel (Figure 9). The leaching degree of Ni was statistically significantly different between *At. caldus* at pH 1.5 and not maintained pH (*p* = 0.012).

**Figure 10:**
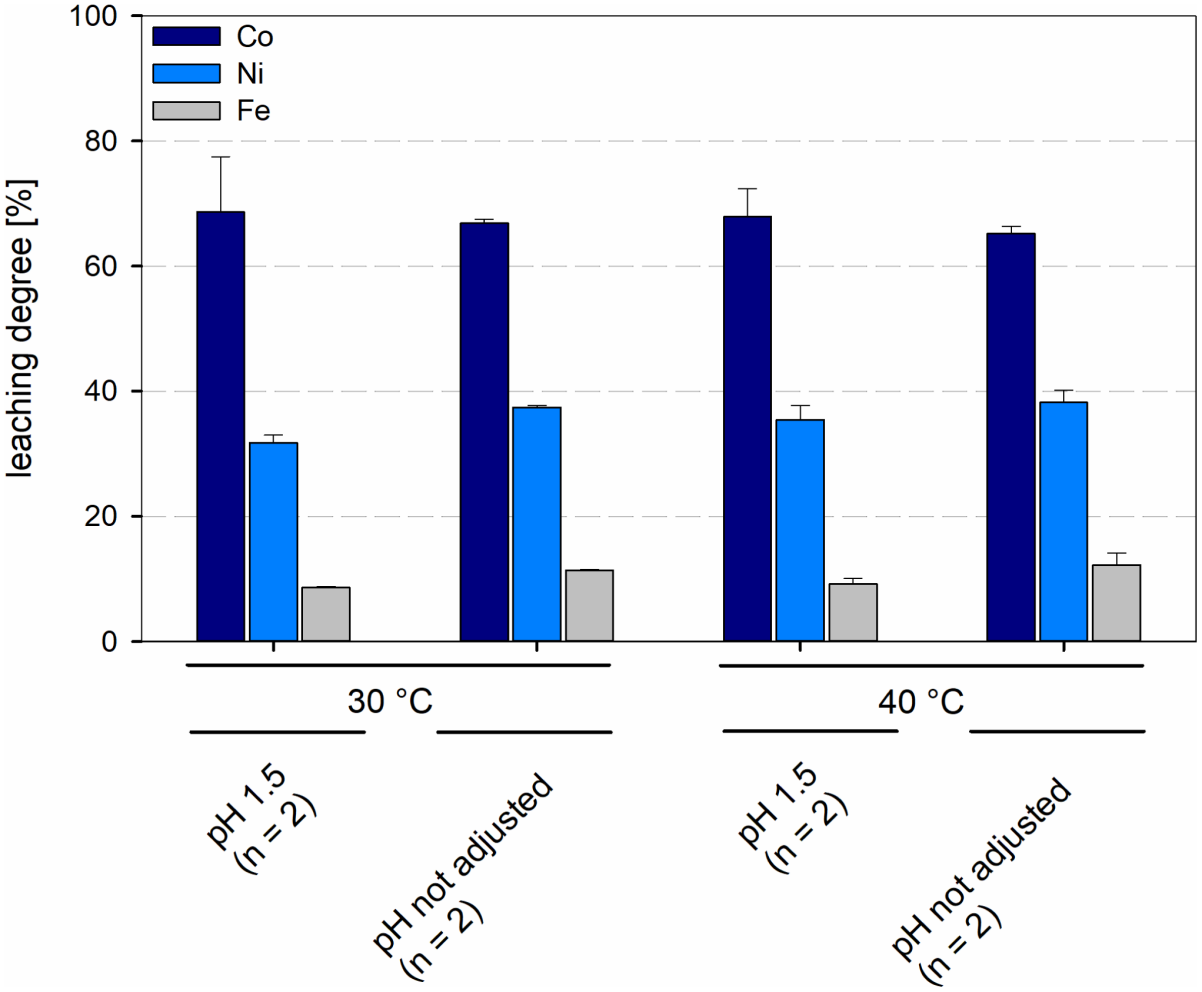
Leaching degree for Co, Ni, and Fe in laterite bioleaching experiments with a mesophilic (30°C) and a moderately thermophilic (40°C) consortium after 15 days under oxic conditions in 2-L stirred-tank reactors at pH 1.5 or not maintained pH. Error bars show standard deviations from the mean values for two parallel bioreactor runs.

In bioreactors with consortia consisting of different acidophilic sulfur- and iron-oxidizers leaching degrees of cobalt and nickel were similar in all reactors, regardless of temperature and pH. Leaching degrees for cobalt and nickel at 30°C were 68.7 ± 8.8% and 31.8 ± 1.2%, respectively, at pH 1.5 and 66.8 ± 0.7% and 37.4 ± 0.3%, respectively, at not maintained pH, with leaching degrees of Ni showing a statistically significant difference (*p* = 0.024) between maintained and not maintained pH for 30°C. Consortia at 40°C and maintained pH 1.5 showed leaching degrees of 67.9 ± 4.5% for cobalt and 35.4 ± 2.3% for nickel, when the pH was not maintained the leaching degrees were 65.2 ± 1.2% for cobalt and 39.6 ± 3.9% for nickel (Figure 10). No statistically significant difference was detected between maintained and not maintained pH for 40°C.

## 4 Discussion

This study confirmed that bioleaching of laterite with acidophilic, sulfur-oxidizing bacteria under oxic conditions is a viable process option. Extraction of nickel and cobalt in stirred-tank reactor bioleaching experiments with the same laterite sample BaSt and pure cultures of acidophiles was 50 and 76%, respectively, for *At. thiooxidans* and 56 and 88%, respectively, for *At. caldus* in a previous study in our laboratory (Stanković et al., 2022); thus, similar values were obtained in this new study. The effect of potential contamination in industrial bioleaching operations with ferrous iron-oxidizing acidophiles active at low pH such as *Leptospirillum* species (Smith and Johnson, 2018) was particularly investigated in a couple of laterite bioleaching experiments with the addition of *Leptospirillum* species as artificial contaminants in stirred-tank reactors. As expected, for both mixed cultures, *At. thiooxidans* with *L. ferrooxidans* at 30°C as well as *At. caldus* with *L. ferriphilum* at 40°C, ferrous iron was efficiently oxidized to ferric iron leading to a small proportion of ferrous iron to total iron and a much higher redox potential than in the bioreactors with the respective pure cultures, *At. thiooxidans* and *At. caldus*. Surprisingly, despite acidophilic ferrous iron-oxidizers, that is, *Leptospirillum* spp., being present and therefore ferrous iron concentrations being low and redox potentials maintained high, leaching of cobalt and nickel was almost as efficient as in pure cultures of sulfur-oxidizing *Acidithiobacillus* spp. under identical conditions in bioreactors. Results imply an efficient reductive dissolution of the laterite mineral phase, despite the concentrations of ferrous iron being barely detectable in mixed cultures of sulfur- and iron-oxidizers (Smith and Johnson, 2018).

In their study using a co-culture of *At. caldus* with *L. ferriphilum* under oxic conditions, Smith and Johnson (2018) stated that ferric iron sensitivity and a high positive redox potential were responsible for lower rates of both sulfur oxidation and growth of *At. caldus*, which might explain the somehow lower but not statistically significant bioleaching efficiency in mixed culture in our experiment. In fact, *At. thiooxidans* might be less sensitive to high ferric iron concentrations, and therefore, the leaching degree was almost the same as in mixed culture with *L. ferrooxidans*.

Overall, the difference in cobalt and nickel extraction was negligible for the different experiments (despite different pH values); thus, the question remains why a dramatically lowered ferrous iron concentration in the presence of *Leptospirillum* spp. did not have a significant effect on the bioleaching of laterite. Ferrous iron is considered to be an efficient reductant for several oxidized cobalt and nickel-bearing mineral phases in laterite throughout bioleaching with acidophiles (Stanković et al., 2022; Moro et al., 2023; Santos and Schippers, 2023). However, during the oxidation of elemental sulfur by acidophiles under oxic conditions ferric iron is likely chemically reduced, probably by sulfur compounds formed during the process as suggested by Marrero et al. (2020). Recently, hydrogen sulfide and thiosulfate were detected as such sulfur intermediates in ferric iron reduction experiments with acidophilic sulfur-oxidizers (Breuker and Schippers, 2024). Such inorganic sulfur compounds would also directly (not only indirectly via iron cycling) reduce mineral phases such as manganese and iron(hydr)oxides (Schippers and Jørgensen, 2001) carrying nickel and cobalt; thus, in fact, these compounds might be even more relevant for reductive laterite bioleaching under oxic conditions than ferrous iron.

Overall, the dissolution of oxide minerals such as goethite in limonitic laterites is based on three processes (Hallberg et al., 2011; Johnson et al., 2021; Vera et al., 2022), which might co-occur during “reductive” bioleaching of laterites with sulfur-oxidizing acidophiles:

1. Microbial acid generation via oxidation of elemental sulfur to sulfuric acid leads to acid leaching.
2. Microbial ferric iron reduction coupled with elemental sulfur oxidation shifts the equilibrium between goethite solid-phase and soluble ferric iron, thereby contributing to goethite dissolution.
3. Microbial release of inorganic sulfur compounds such as hydrogen sulfide serving as a chemical reductant of oxide minerals.

Conclusively, despite the presence of ferrous iron-oxidizing acidophiles (*Leptospirillum* spp.) significantly reducing the ferrous iron concentration, no significant decrease in the extraction rates of cobalt and nickel from a laterite ore occurred using bioleaching with sulfur-oxidizing acidophiles (*Acidithiobacillus* spp.) under oxic conditions. Therefore, bioleaching of laterite under oxic conditions remains a suitable process option.

## Data availability statement

The raw data supporting the conclusions of this article will be made available by the authors, without undue reservation.

## Author contributions

SH: Investigation, Methodology, Formal analysis, Validation, Writing – original draft. AS: Conceptualization, Funding aquisition, Project administration, Writing – review & editing.
